# Advancing precision oncology with AI-powered genomic analysis

**DOI:** 10.3389/fphar.2025.1591696

**Published:** 2025-04-30

**Authors:** Ruby Srivastava

**Affiliations:** Bioinformatics, Centre for Cellular and Molecular Biology-CSIR, Hyderabad, India

**Keywords:** genomics, precision oncology, bioinformatics, artificial intelligence, therapeutics and analysis resource

## Abstract

Multiomics data integration approaches offer a comprehensive functional understanding of biological systems, with significant applications in disease therapeutics. However, the quantitative integration of multiomics data presents a complex challenge, requiring highly specialized computational methods. By providing deep insights into disease-associated molecular mechanisms, multiomics facilitates precision medicine by accounting for individual omics profiles, enabling early disease detection and prevention, aiding biomarker discovery for diagnosis, prognosis, and treatment monitoring, and identifying molecular targets for innovative drug development or the repurposing of existing therapies. AI-driven bioinformatics plays a crucial role in multiomics by computing scores to prioritize available drugs, assisting clinicians in selecting optimal treatments. This review will explain the potential of AI and multiomics data integration for disease understanding and therapeutics. It highlight the challenges in quantitative integration of diverse omics data and clinical workflows involving AI in cancer genomics, addressing the ethical and privacy concerns related to AI-driven applications in oncology. The scope of this text is broad yet focused, providing readers with a comprehensive overview of how AI-powered bioinformatics and integrative multiomics approaches are transforming precision oncology. Understanding bioinformatics in Genomics, it explore the integrative multiomics strategies for drug selection, genome profiling and tumor clonality analysis with clinical application of drug prioritization tools, addressing the technical, ethical, and practical hurdles in deploying AI-driven genomics tools.

## 1 Introduction

The emergence of advanced and cost-effective high-throughput technologies (Misra et al., 2018) has generated vast amounts of biological data, ushering in a new era of precision medicine in oncology (Srivastava, 2023a). Precision medicine offers significant potential for cancer treatment and management, enabling oncologists to tailor therapies for individual patients. Precision oncology focuses on treating specific groups of cancer patients by utilizing population-specific diagnostic or prognostic biomarkers. This information is crucial for monitoring disease progression and assessing a patient’s response to treatment. Additionally, it helps identify the molecular mechanisms underlying drug resistance, allowing for the targeted inhibition of genes or pathways responsible for resistance. Precision medicine relies on large datasets that must be processed and analysed to detect molecular patterns and make patient-specific treatment decisions. However, handling these extensive datasets is both costly and time-consuming, a challenge exacerbated by the continuous growth of data due to high-throughput technologies (Misra et al., 2018; Ahmed, 2020; Srivastava, 2024a; Srivastava, 2024b).

Artificial intelligence (AI) and machine learning (ML) provide solutions to these challenges. AI encompasses a range of machine-driven functions, including rule-based logic, machine learning (ML), deep learning (DL), natural language processing (NLP), and computer imaging (Srivastava, 2023a; Ahmed, 2020). The rapid advancement of technologies capable of generating vast amounts of omics data—such as genomic, transcriptomic, proteomic (phenotypic), and epigenomic data—has underscored the necessity of AI in medical data analysis. The surge in genomic and transcriptomic data is primarily attributed to next-generation sequencing (NGS), while the increase in proteomic data results from mass spectrometric analysis (Srivastava, 2023a; Srivastava, 2024a; Srivastava, 2024b). AI can also predict the impact of genetic mutations on protein structure and function. Treatment efficacy and adverse effects can vary based on factors such as age, sex, genetics, and environmental influences, including anthropometric and metabolic status, dietary patterns, and lifestyle choices (Jaccard et al., 2018). Precision medicine aims to design the most effective interventions based on an individual’s biological profile (Tebani et al., 2016). Clinical data and omics information can be obtained from databases or collected through screening technologies for various purposes, including disease diagnosis (Menyhárt and Gyorffy, 2021), class prediction (Hasin et al., 2017), biomarker discovery (Sun and Hu, 2016), disease subtyping (Menyhárt and Gyorffy, 2021), enhanced systems biology insights (Dahal et al., 2020), and drug repurposing (Srivastava, 2022; Srivastava, 2023b).

Multiomics refers to the comprehensive analysis of multiple layers of biological data such as genomics (DNA), transcriptomics (RNA), proteomics (proteins), epigenomics (epigenetic modifications), and metabolomics (metabolites) to gain a holistic understanding of biological systems (Chen et al., 2023). Integrating these diverse omics datasets is crucial for precision oncology because cancer is a complex, multi-factorial disease involving alterations at various molecular levels. By combining insights from different omics layers, researchers and clinicians can uncover more accurate biomarkers, better understand tumor heterogeneity, and identify personalized therapeutic targets, ultimately leading to more effective, tailored cancer treatments (Correa-Aguila et al., 2022). The current applications of AI in multiomics data analysis, emphasizes its role in precision oncology and therapeutics. Transcriptomics research has provided insights into the molecular mechanisms underlying both physiological processes (e.g., developmental stages, cell cycle phases) and pathological conditions, leading to clinical applications such as MammaPrint^®^, a 70-gene panel used to predict the risk of relapse and metastasis in breast cancer (Hamet and Tremblay, 2017). While single-omics analyses have contributed valuable findings, many prevalent diseases with high mortality rates, such as type 2 diabetes and cardiovascular disease, still lack effective therapeutic solutions (Hamet and Tremblay, 2017). This is partly because the functions of genetic variants are not always easily interpretable, often limiting the development of targeted treatments.

The human genome consists of approximately 3 billion base pairs, encompassing both coding and non-coding regions (Makałowski, 2001). A key distinction within the genome is the difference between introns and exons—introns are non-coding segments of genes, whereas exons represent coding regions responsible for protein production. Whole Genome Sequencing (WGS) is a comprehensive technique that sequences an organism’s entire DNA, allowing for the identification of genetic variants and providing a complete picture of genomic composition. As mentioned earlier, genomics research focuses on genetic variations such as single nucleotide polymorphisms (SNPs) and larger structural changes that contribute to an organism’s genetic makeup. SNPs are the most prevalent form of genetic variation, representing differences in a single nucleotide (Nakagawa et al., 2015; Nakagawa and Fujita, 2018).

Genomic alterations affecting large DNA segments (≥50 bp) are classified as structural variations (SVs). These variations arise from multiple mutational mechanisms, including deletions, insertions, and duplications, which alter the genomic sequence quantity and are collectively referred to as copy-number variations (CNVs) (Escaramıs et al., 2015; Li et al., 2020; Ho et al., 2020). Various techniques have been employed to study CNVs, with Whole Genome Sequencing (WGS) increasingly emerging as the preferred approach due to its declining costs and continuous improvements in variant detection methods (Pos et al., 2021).

CNVs can be analyzed using WGS by identifying genomic regions with an abnormal number of sequencing reads compared to expected levels, a method known as depth of coverage (DOC) analysis. Given its ability to yield reliable results even at shallow sequencing depths (0.19–1.09 coverage of the genome), CNV analysis has become a valuable tool in clinical diagnostics (Dong et al., 2017) Several tools, such as WISECONDORX, have been developed to characterize CNVs and assess their clinical and therapeutic significance (Raman et al., 2019).

While WGS sequences all DNA, including both coding and non-coding regions, Whole Exome Sequencing (WES) specifically targets protein-coding regions of genes (Lelieveld et al., 2015). WES selectively sequences these regions along with approximately 20 nucleotides of adjacent intronic sequences to investigate protein-coding areas in greater detail. The field of genomics was first introduced by American geneticists in 1986 to study the composition, structure, function, localization, and editing of DNA. Today, genomics is used to analyze all genes within an organism, providing insights into their biological significance. Advancements in genomic technologies have made it possible to efficiently analyze whole-genome data, leading to the discovery of genes, proteins, and biological pathways associated with diseases. In drug-target screening, genomic technologies compare DNA sequencing data from tumor and non-malignant tissues to identify key genetic differences. These differential genes can serve as potential drug targets and can be further validated using CRISPR-Cas9 knockout technology, allowing researchers to individually screen and assess their impact (Chan et al., 2022; Yamamoto et al., 2019). Genomic research is divided into three primary areas: structural genomics, functional genomics, and comparative genomics. Structural genomics focuses on analyzing nucleotide sequences through whole-genome sequencing to determine genome composition and gene positioning. Functional genomics involves modifying gene sequences or their expression within cells to observe resulting phenotypic changes, thereby linking genotype to phenotype and clarifying gene functions. Comparative genomics examines variations in genome structure and function across different species to understand their evolutionary and biological relationships (Haley and Roudnicky, 2020). Functional genomics, which explores gene functions and networks, has become a crucial tool for understanding the complex interactions within human tumors and their microenvironments. Technologies such as RNA interference (Yin and Kassner, 2016; Adams et al., 2016), small interfering RNA (siRNA) (Zhang et al., 2007), short hairpin RNA (shRNA) (Takase et al., 2017), CRISPR interference, and CRISPR inhibition (le Sage et al., 2017; DePristo et al., 2011) are instrumental in drug-target discovery and validation. Bioinformatics plays a critical role in analyzing cancer somatic mutations, as these mutations are key targets for precision therapies that minimize damage to healthy cells. However, germline variants, which influence drug metabolism and substrate interactions, can significantly impact drug efficacy and toxicity. Therefore, considering both somatic and germline variations is essential when developing personalized treatment strategies (Koboldt, 2020).

The performance of different AI algorithms as deep learning (DL) and traditional machine learning (ML) in multi-omics data analysis has been extensively evaluated across various studies, each offering distinct strengths and limitations depending on the nature of the task and dataset (Wei et al., 2023). Traditional machine learning algorithms such as random forests, support vector machines (SVMs), k-nearest neighbors (KNN), and gradient boosting methods (e.g., XGBoost) have been widely used due to their robustness, ease of implementation, and interpretability. These models typically perform well in structured, relatively low-dimensional, and properly preprocessed datasets (Sarker, 2021). They are particularly effective when individual omics layers are analyzed separately or integrated in a relatively simple manner (e.g., feature concatenation). However, their performance may plateau when dealing with complex, high-dimensional multi-omics datasets due to limited capacity to capture nonlinear relationships and cross-omics interactions (Subramanian et al., 2020). In contrast, deep learning algorithms such as convolutional neural networks (CNNs), recurrent neural networks (RNNs), autoencoders, variational autoencoders (VAEs), and graph neural networks (GNNs) excel in modeling complex and high-dimensional multi-omics data (Alzubaidi et al., 2021). DL models are particularly well-suited for capturing intricate, nonlinear interactions between various omics layers (genomics, transcriptomics, proteomics, etc.), making them powerful tools for tasks such as disease classification, biomarker discovery, and survival prediction (Nguyen PHD. et al., 2021). For example, multi-modal deep learning frameworks that use multi-omics integration techniques like late fusion, intermediate fusion, or attention mechanisms often outperform traditional ML approaches in predictive performance (Nakach et al., 2024). However, deep learning requires large amounts of data to avoid overfitting and due to its limited interpretability, it generate significant drawback in clinical contexts. Moreover, ensemble and hybrid approaches which combine both ML and DL techniques are increasingly used to leverage the strengths of each method (Almulihi et al., 2022). For instance, feature representations extracted from deep learning models can be fed into ML classifiers for improved performance and interpretability. Emerging trends also emphasize explainable AI (XAI) to improve the transparency of deep learning models in biomedical applications (Mathew et al., 2025).

The effectiveness of different AI algorithms particularly deep learning (DL) versus traditional machine learning (ML) in analyzing multiomics data across various types of cancer varies depending on cancer type, data complexity, and clinical endpoints. Each algorithm has unique strengths that make it more suitable for certain scenarios in cancer research (Arjmand et al., 2022). In breast cancer, where extensive multiomics datasets (e.g., genomics, transcriptomics, proteomics, and methylation data) are available from sources like TCGA (Tomczak et al, 2015), DL models such as autoencoders and multimodal neural networks have demonstrated superior performance in tasks like subtype classification, survival prediction, and treatment response modeling (Yang et al., 2024). DL excels due to its ability to capture complex nonlinear interactions among heterogeneous data layers. However, ML algorithms like random forests and SVMs still perform competitively when applied to well-engineered features, offering higher interpretability and lower computational costs. In lung cancer, particularly non-small cell lung cancer (NSCLC), ensemble ML methods (e.g., XGBoost and random forests) have shown strong predictive performance in biomarker discovery and prognosis prediction, especially when integrating genomics with transcriptomics or imaging data (Li et al., 2022). DL methods, especially graph neural networks (GNNs), have become effective in modeling protein–protein interaction networks or pathway-level features but are limited by smaller cohort sizes in some datasets. In glioblastoma multiforme (GBM), a highly heterogeneous brain tumor, DL particularly variational autoencoders and multi-view DL architectures outperforms traditional ML in integrating multiomics data (e.g., methylation, copy number variation, transcriptomics) for subtype discovery and survival prediction (Poursaeed et al., 2024). DL’s capacity to learn latent representations from noisy, high-dimensional data is especially beneficial in such complex cancers. For colorectal and prostate cancers, where the multiomics landscape is less characterized than breast or lung cancer, ML algorithms are often preferred due to smaller sample sizes. Random forests and logistic regression are widely used for classification and feature selection, especially in studies focused on diagnostic and prognostic biomarker discovery (Wei et al., 2022; Hachem et al., 2024; Bao et al., 2024). In pan-cancer studies, where multiomics data across multiple tumor types are analyzed collectively, DL models like multimodal neural networks and transformers have emerged as powerful tools for learning shared and distinct features across cancers, enabling cross-cancer subtype clustering and drug response prediction. However, pan-cancer DL models require large, well-curated datasets and advanced techniques to avoid biases introduced by imbalanced data distributions across cancer types (Divate et al., 2022). A hybrid approach, combining the representation power of DL with the interpretability of ML, is increasingly adopted to leverage the strengths of both in cancer multiomics research (Mavaie et al., 2023).

## 2 Bioinformatics approaches in genomics

Genomic data analysis involves examining single nucleotide polymorphisms (SNPs), copy number variations, gene expression, microRNA expression, protein expression, and other genetic alterations. Precision oncology leverages high-throughput technologies and bioinformatics tools to personalize cancer treatments based on individual genetic profiles. This approach enhances the ability to identify and validate biomarkers crucial for cancer diagnosis, prognosis, and tailored therapeutic strategies. Successfully integrating bioinformatics into precision oncology requires expertise in oncology, bioinformatics, and biostatistics (Li et al., 2018; Szymczak et al., 2009). Whole Genome Sequencing and Whole Exome Sequencing enable rapid, comprehensive analysis of genetic mutations, SNPs, and structural variations within tumors.

Bioinformatics tools play a key role in data integration and interpretation, particularly in variant annotation and functional prediction. Tools like ANNOVAR (Wang et al., 2010) facilitate the identification of actionable mutations by providing scores from multiple predictive models, including SIFT (Ng and SIFT, 2003), PolyPhen-2 (Adzhubei et al., 2013), LRT (Chen et al., 2020), FATHMM (Rogers et al., 2018), MetaSVM and MetaLR (Dong et al. 2015), VEST3 (Carter et al., 2013), and CADD (Kircher et al. 2014). SIFT (Ng and SIFT, 2003) determines whether a variant is deleterious by using PSI-BLAST to assess amino acid conservation across closely related sequences. PolyPhen-2 (Adzhubei et al., 2013) employs a pipeline combining eight sequence-based and three structure-based methods to classify mutations as benign, probably deleterious, or known to be deleterious. The Likelihood Ratio Test (LRT) (Chen et al., 2020) evaluates conservation across closely related species to determine the functional impact of mutations. FATHMM (Rogers et al., 2018) utilizes Hidden Markov Models and sequence conservation to predict the effects of missense mutations on protein function. MetaSVM and MetaLR are ensemble methods that integrate ten predictor scores (SIFT, PolyPhen-2 HDIV, PolyPhen-2 HVAR, GERP++, MutationTaster, Mutation Assessor, FATHMM, LRT, SiPhy, and PhyloP) along with the maximum observed frequency from the 1,000 Genomes Project to predict deleterious variants. MetaSVM is based on Support Vector Machines (SVM), while MetaLR employs Logistic Regression (LR) to generate final variant scores.

Pathway analysis tools, such as Ingenuity Pathway Analysis (IPA) (Krämer et al., 2014) and Gene Set Enrichment Analysis (GSEA) (Mootha et al., 2003), play a crucial role in identifying disrupted biological pathways and networks, offering valuable insights into tumorigenesis. Bioinformatics has been instrumental in pinpointing immunotherapy targets like Programmed Death Ligand 1 (PD-L1) (Han et al., 2020) and in identifying biomarkers for epidermal growth factor receptor (EGFR) inhibitors in non-small cell lung cancer (Prabhakar, 2015), as well as poly (ADP-ribose) polymerase (PARP) inhibitors for cancers with Breast Cancer Gene 1/2 (BRCA1/2) mutations (Faraoni and Graziani, 2018). These biomarkers undergo rigorous validation to ensure their accuracy and clinical relevance.

As biomarker identification techniques improve and genomic data repositories continue to expand, the sheer complexity and volume of data necessitate more sophisticated analytical tools. This growing demand has fueled the increasing reliance on machine learning (ML) and predictive algorithms. ML techniques excel at handling large, high-dimensional datasets, uncovering patterns, and establishing relationships within the data. By employing dimensionality reduction and feature selection, ML algorithms enhance the efficiency of biological data analysis, allowing for the evaluation of disease mechanisms and the identification of potential biomarkers. To enhance cancer patient care, precision treatment should include monitoring and managing Quality of Life (QoL) data collected in the patient’s home environment, along with its integration and analysis. Recent advanced technologies has facilitated the development of smartphone devices that support both patients and clinicians by consolidating all relevant patient data and assisting with patient-reported outcomes (Srivastava, 2023b; Srivastava, 2023c). Genome-wide association studies (GWAS) have also generated vast amounts of genomic data for cancer research. The successful application of patient-specific data in precision medicine hinges on the accurate integration, analysis, and interpretation of these datasets to provide a comprehensive overview of gene expression changes in individual cancer patients (Li et al., 2018; Szymczak et al., 2009; Telenti et al., 2018). Such analyses can reveal alterations in metabolic and signaling pathways specific to a patient, paving the way for highly personalized treatment plans. This multidimensional approach offers significant advantages over traditional single-layer analyses, which focus on isolated features (Chari et al., 2010; Wang et al., 2014). However, for AI to effectively interpret such data, it must first be trained to recognize key features and patterns.

Over the past decade, large-scale cancer research initiatives have emerged to streamline the analysis of omics data. Projects such as The Cancer Genome Atlas (TCGA) [27], the International Cancer Genome Consortium (ICGC) (ICGC, 2022 Hudson et al., 2010), COSMIC (Tate et al., 2019), TARGET, and the German Cancer Consortium (DKTK) (Joos et al., 2019), along with platforms like the Genomic Data Commons (GDC) [32], cBioPortal (Cerami et al., 2012), UCSC Genome Browser (Rosenbloom et al., 2013), Array Express (Parkinson et al., 2007), and Gene Expression Omnibus (GEO), have significantly contributed to this effort. The list of various databases, their links and types of data analyses of human tumors and tumor cell lines are given in Table 1.

**TABLE 1:** The list of various databases, their links and types of data analyses of human tumors and tumor cell lines.

SN	Database	Link	Types of data/analyses
1.	TCGA	https://www.cancer.gov/ccg/research/genome-sequencing/tcga	20,000 primary cancer and matched normal samples spanning 33 cancer types
2.	ICGC-AGRO	https://www.icgc-argo.org/	ICGC ARGO will uniformly analyze specimens from 100,000 cancer patients
3.	GDC	https://portal.gdc.cancer.gov/	Explore and analyse clinical and genomic data from cancer genomics studies
4.	GEO	https://www.ncbi.nlm.nih.gov/geo/	GEO is a public functional genomics data repository supporting MIAME-compliant data submissions.
5.	Array Express	https://www.ebi.ac.uk/biostudies/arrayexpress	Stores high-throughput genomics data.
6.	ENA	https://www.ebi.ac.uk/ena/browser/home	Stores raw data files in Fastq format.
7.	Sequence Read Archive	https://www.ncbi.nlm.nih.gov/sra	Stores raw data files in SRA format.
8.	Dryad Digital Repository	https://datadryad.org/stash	Open access repository of medical research data
9.	Figshare	https://figshare.com/	Cross-disciplinary open-access repository for academic research
10.	Harvard Dataverse Network	https://dataverse.harvard.edu/	Multi-disciplinary data storage center
11.	Kaggle	https://www.kaggle.com/	Platform for data science training, competitions, and datasets
12.	Network Data Exchange	https://home.ndexbio.org/about-ndex/	Repository for network biology data
13.	Open Science Framework	https://osf.io/	Platform for collaborating on research projects
14.	GenoVault	https://github.com/bioinformaticscdac/GenoVault	Cloud-based repository for NGS data
15.	UK Biobank	https://www.ukbiobank.ac.uk/	Large-scale biomedical research database
16.	cBioPortal	https://www.cBioPortal.org/	Visualizations, analysis, cancer genomics projects
17.	COSMIC	https://cancer.sanger.ac.uk/cosmic	Database of somatic mutations in cancer.
18.	IGV	https://software.broadinstitute.org/software/igv/	High-performance genome browser for visualizing and analyzing large-scale genomic data.
19.	Regulome Explorer	https://explorer-cancerregulome.systemsbiology.net/	Exploring and analyzing regulatory elements in the genome.
20.	UCSC Genome Browser	https://genome.ucsc.edu/	Provides access to a vast collection of genomic data and annotations
21.	Bioconductor	https://www.bioconductor.org/	Open-source software project for the analysis and comprehension of high-throughput genomics data.
22.	Cytoscape	https://cytoscape.org/	Network analysis and visualization tool
23.	Gene Ontology	http://geneontology.org/	Standardized system for annotating genes and their functions in different organisms.
24.	UALCAN	https://ualcan.path.uab.edu/	Web portal for in-depth analysis of cancer transcriptome data.
25.	DAVID	https://david.ncifcrf.gov/	Functional annotation and enrichment analysis of gene lists
26.	HumanBase (GIANT)	https://hb.flatironinstitute.org/	Exploring human genomic data and conducting large-scale integrative analysis.
27.	CEDER	https://pmc.ncbi.nlm.nih.gov/articles/PMC3488134/	Detection of differentially expressed genes
28.	CPTRA	https://gdc.cancer.gov/about-gdc/contributed-genomic-data-cancer-research/clinical-proteomic-tumor-analysis-consortium-cptac	Package for analyzing transcriptome sequencing data
29.	Bioconductor	https://www.bioconductor.org/	Open-source software for genomic data analysis
30.	LIMMA	https://bioconductor.org/packages/release/bioc/html/limma.html	Statistical package for the analysis of microarray and RNA-seq data.
31.	CARET	https://cran.r-project.org/web/packages/caret/index.html	R package for training and evaluating ML models.
32.	netClass	https://doi.org/10.1093/bioinformatics/btu025	A tool for classifying biological samples using network-based features.
33.	WGCNA	https://horvath.genetics.ucla.edu/html/CoexpressionNetwork/Rpackages/WGCNA/	Identifying gene modules and their relationships in high-throughput data.
34.	MyCancerGenome	https://www.mycancergenome.org/	Understanding cancer genomics and personalized cancer treatment options.
35.	CIViC	https://civicdb.org/welcome	Treatment options for cancer patients based on their unique tumor DNA Molecular characterization
36.	TARGET	https://www.cancer.gov/ccg/research/genome-sequencing/target	Molecular characterization
37.	CGI	https://www.genomicinterpretation.org/	Genomic alterations in cancer and their potential clinical relevance
38.	ClinicalTrials.gov	https://www.clinicaltrials.gov/	An online database that provides information on clinical trials
39.	EUCTR	https://www.clinicaltrialsregister.eu/	Database containing information on clinical trials conducted in the European Union
40.	OncoKB	https://www.oncokb.org/	Mutations, CNVs, fusions
41.	DepMap	https://depmap.org/portal/	Genetic loss-of-function screening, pharmacologic dependencies, CCLE omics characterizations
42.	canSAR.ai	https://cansar.ai/	Integrates biology, chemistry, pharmacology, structural biology, cellular networks and clinical annotations, and applies machine learning approaches to develop predictions useful in drug discovery
43.	ClinGen	https://clinicalgenome.org/	Clinical relevance of genes and variants
44.	ClinVar	www.ncbi.nlm.nih.gov/clinvar/intro/	Database of genomic variants with public submissions of variant interpretations and disease relations.
45.	Cosmic	https://cancer.sanger.ac.uk/cosmic	Catalogue Of Somatic Mutations In Cancer
46.	dbSNP	https://www.ncbi.nlm.nih.gov/snp/	Contains human single nucleotide variations, microsatellites, and small-scale insertions and deletions
47.	Ensembl	https://www.ensembl.org/index.html	Genome browser for vertebrate genomes that supports research in comparative genomics, evolution, sequence variation and transcriptional regulation
48.	Find Zebra	https://www.findzebra.com/	Tool for helping diagnosis of rare diseases. It uses freely available high quality curated information on rare diseases
49.	Genomics England	https://www.genomicsengland.co.uk/	Comprehensive site describing the progress of the UK sequencing initiative. Site contains usefull overviews over gene panels and diseases.
50.	gnomAD	https://gnomad.broadinstitute.org	Exome and genome sequencing data with allele frequencies from a wide variety of large-scale sequencing projects
51.	GTEX	https://gtexportal.org/home/	Comprehensive public resource to study tissue-specific gene expression and regulation. Samples were collected from 54 non-diseased tissue sites across nearly 1000 individuals, primarily for molecular assays including WGS, WES, and RNA-Seq.
52.	HGMD	https://www.hgmd.cf.ac.uk/ac/index.php	Collate all known (published) gene lesions responsible for human inherited disease
53.	Human Phenotype Ontology (HPO)	https://hpo.jax.org/app/	Provides a standardized vocabulary of phenotypic abnormalities encountered in human disease
54.	Matchmaker Exchange	https://www.matchmakerexchange.org	Genomic discovery through the exchange of phenotypic and genotypic profiles
55.	MaveDB	https://www.mavedb.org/	Collection, distribution, and analysis of variant effect maps
56.	MedGen	https://www.ncbi.nlm.nih.gov/medgen/	Organizes information related to human medical genetics, such as attributes of conditions with a genetic contribution
57.	NCBI	https://www.ncbi.nlm.nih.gov/	The National Center for Biotechnology Information advances science and health by providing access to biomedical and genomic information
58.	OMIM	https://www.omim.org/	Compendium of human genes and genetic phenotypes
59.	RefSeq	https://www.ncbi.nlm.nih.gov/refseq/	A comprehensive, integrated, non-redundant, well-annotated set of reference sequences including genomic, transcript, and protein.
60.	Uniprot	https://www.uniprot.org/	Comprehensive and freely accessible resource of protein sequence and functional information.
61.	RefSeq	https://www.ncbi.nlm.nih.gov/refseq/	A comprehensive, integrated, non-redundant, well-annotated set of reference sequences including genomic, transcript, and protein.

Abbreviations: TCGA, The Cancer Genome Atlas Program; ICGCAGRO, The International Cancer Genome Consortium Accelerating Research in Genomic Oncology; GDC, Genomic Data Commons; GEO, Gene Expression Omnibus; ENA, The European Nucleotide Archive; COSMIC, Catalogue Of Somatic Mutations In Cancer; IGV, Integrative Genome Viewer; UALCAN, University of Alabama at Birmingham Cancer Data Analysis Portal; DAVID, Database for Annotation, Visualization, and Integrated Discovery; CEDER, Cancer Epitope Database and Analysis Resource; CPTRA, cross-platform transcriptome analysis; Limma, Linear Models for Microarray and Omics Data; CARET, Carotene and Retinol Efficacy TrialCarotene and Retinol Efficacy Trial; CIViC, Clinical Interpretation of Variants in Cancer; TARGET, *Therapeutically Applicable Research to Generate Effective Treatments*; WGCNA, Weighted correlation network analysis; CGI, Cancer Genome Interpreter; EUCTR, EU Clinical Trials Register; GTEx, Genotype-Tissue Expression; HGMD, Human Gene Mutation Database; NCBI, The National Center for Biotechnology Information, OMIM, *Online Mendelian Inheritance in Man*; *CCLE*, *Cancer Cell Line Encyclop*edia; CNV, copy-number variation; DepMap, Dependency Map.

For instance, a study on lung adenocarcinoma by Gillette et al. (2020) [37] refined tumor classification by dividing the proximal-proliferative cluster using transcriptomic data, deep-scale proteomic profiling, and post-translational modifications. Despite these advancements, several challenges persist in integrative analysis, including (i) the high dimensionality of data, which complicates inference; (ii) inherent heterogeneity across different technical platforms, reducing biological signal clarity; (iii) the diversity of data types, making it unlikely that a single analytical method will be applicable across all omics layers; and (iv) the difficulty of interpretation due to the sheer volume of information, which can obscure meaningful conclusions. To address these challenges, various integrative approaches have been developed, focusing on (i) patient stratification, (ii) clinical outcome prediction, and (iii) identifying molecular mechanisms that operate across different biological layers (Srivastava, 2024a; Srivastava, 2024b). Recent studies have applied causal inference techniques to either validate existing biological relationships (Dugourd et al., 2021) or infer stable connections across multiple experimental conditions without prior knowledge (Meinshausen et al., 2016). Different classification frameworks have been proposed based on application type (unsupervised vs. supervised, with the latter further divided into predictive and explanatory models), strategy (early, intermediate, or late integration), and methodology. The literature commonly categorizes six major families of integrative methods: matrix factorization, Bayesian approaches, multiple kernel learning, ensemble learning, deep learning, and network-based methods (Bersanelli et al., 2016; Huang et al., 2017; Krassowski et al., 2020; Vahabi and Michailidis, 2022; Picard et al., 2021; Subramanian et al., 2020; Broad Institute, 2018).

**FIGURE 1:**
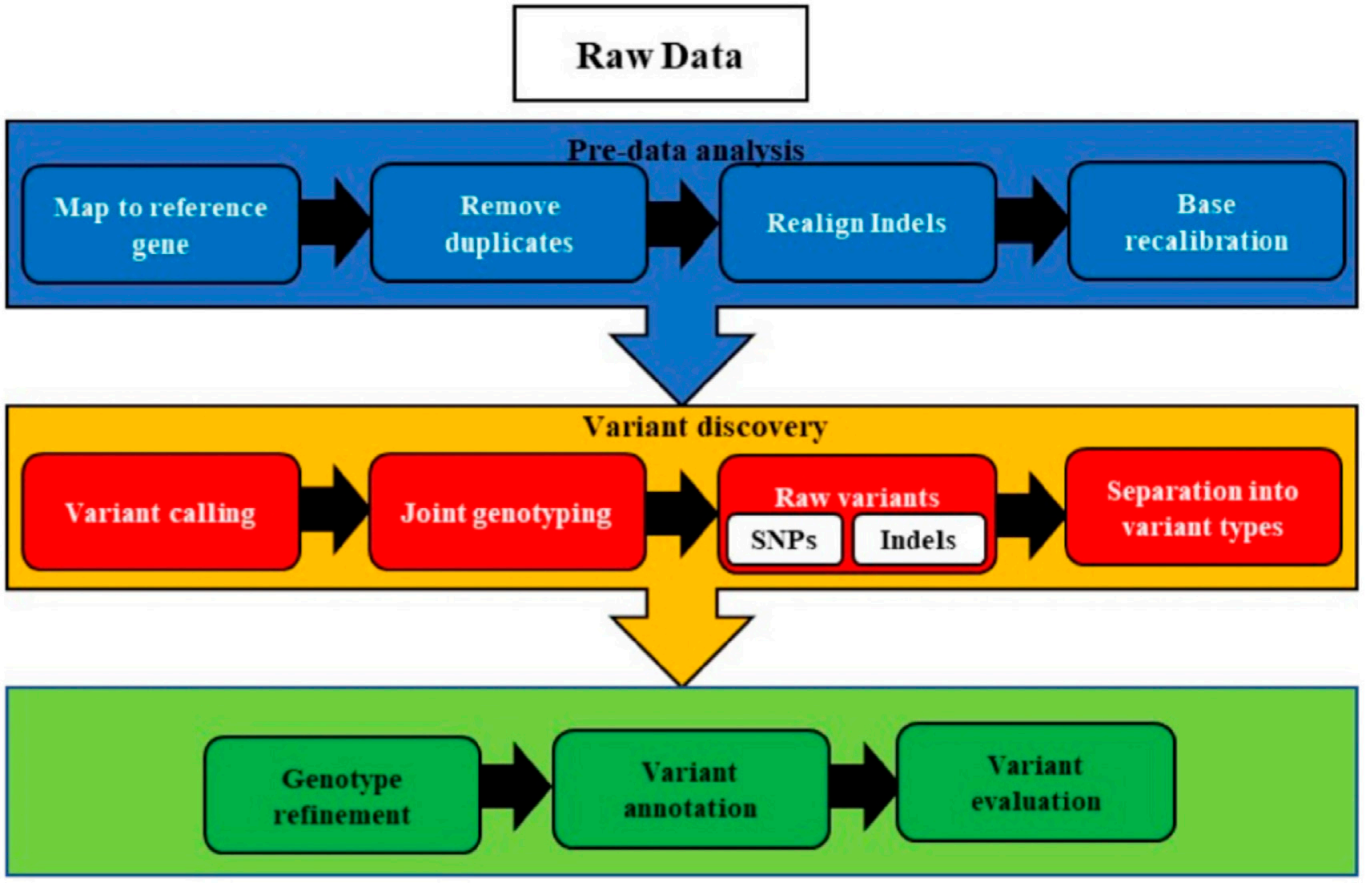
The pre-processing, variants identification, classifications, and comparison to known variants during the raw variant step and sorting of identified variants on specific criteria. Next, the accuracy of the data is enhanced before the full annotation and evaluation of variants.

The bioinformatics pipeline developed and implemented at the Utah Public Health Laboratory (UPHL) consists of eight key steps: (1) read quality control, (2) reference strain identification, (3) read mapping to the reference strain, (4) detection of single nucleotide polymorphisms and small insertions or deletions (INDELs), (5) *de novo* genome assembly, (6) genome annotation, (7) phylogenetic tree construction, and (8) phylogenetic analysis. While these processes are standard, multiple software tools are available to perform each step (Srivastava, 2023b). The first step in genomics-based drug selection involves identifying clinically relevant alterations in cancer patients through variant calling analysis. According to the Genome Analysis Toolkit (GATK) (DePristo et al., 2011), the general workflow of variant calling includes nine steps: quality control (QC) and trimming, alignment, marking duplicates, local realignment of INDELs, base quality score recalibration (BQSR), variant calling, filtering, and annotation of variants (Koboldt, 2020). The basic steps for application of bioinformatics in whole genome sequencing are given in Figure 1.

Following sample QC and trimming, raw sequencing reads are aligned to the reference genome using tools like BWA-MEM (Li, 2013). Duplicate reads are then removed with PICARD. GATK tools are employed to minimize alignment artifacts and improve sequencing quality estimates. Variant calling is performed using tools such as MUTECT2, HAPLOTYPECALLER (McKenna et al., 2010), VARSCAN 2 (Koboldt et al., 2012), VARDICT (Lai et al., 2016), or SOMATICSNIPER (Larson et al., 2012), which identify short variants, including single nucleotide variants (SNVs) and insertions or deletions (INDELs) of less than 50 base pairs (bp). The identified variants undergo filtering to eliminate low-quality calls, followed by annotation to determine their biological impact, population frequency, and clinical relevance. This analysis primarily focuses on somatic variants in coding regions. Nonsynonymous SNVs are considered more detrimental, as they alter the final protein sequence, potentially affecting its folding and function (Sun et at., 2019).

Somatic genomic alterations are classified based on their population frequency as either rare variants or polymorphisms. Variants with a high frequency (>1%) are generally deemed clinically benign. In most patients, at least one detected somatic alteration holds clinical significance (Bieg-Bourne et al., 2017; Sanchez-Vega et al., 2018), as it may influence gene function, suggest preventive surveillance, aid in diagnosis, impact prognosis, or guide treatment selection. Several automated variant annotation tools exist to streamline this process. SNPEFF (Cingolani et al., 2012) assesses the biological impact of candidate variants, while ANNOVAR (Wang et al., 2021) and the VARIANT EFFECT PREDICTOR (VEP) (McLaren et al., 2016) provide additional information on variant population frequency. The Variant Caller with Multinomial Probabilistic Model (VCMM) detects SNVs and INDELs from whole exome sequencing (WES) and whole genome sequencing (WGS) studies by using a multinomial probabilistic model with quality score and strand bias filters. VCMM reduces false-positive and false-negative variant calls compared to GATK and SAMtools, improving the accuracy of variant detection (Shigemizu et al., 2013).

Public data repositories provide valuable resources for annotating candidate somatic variants by linking them to drugs and their interconnections. Some key databases include ClinVar (Landrum et al., 2018), which catalogs genetic variants and their clinical significance; the Catalogue of Somatic Mutations in Cancer (COSMIC) (Tate et al., 2019), which compiles information on the impact of somatic mutations in cancer; OncoKB (Chakravarty et al., 2017) and CIViC (Griffith et al., 2017), which associate somatic cancer variants with clinical and therapeutic implications; and DGIdb (Cotto et al., 2018), a database of gene–drug interactions. See Table 1. These patient-centered tools analyze somatic variants in tumors and can be categorized based on the type of input data required. For instance, with a list of available variants, resources like MTB-REPORT (Perera-Bel et al., 2018), the Cancer Genome Interpreter (CGI) (Tamborero et al., 2018), the Variant Interpretation for Cancer Consortium Meta-Knowledgebase (VICC METAKB) (Wagner et al., 2020), PREMEDKB (Yu et al., 2019), and the SMART Cancer Navigator (Warner et al., 2018) can be useful. Some tools also accept disease or drug-related queries. If a variant calling file (VCF) is available, platforms such as MTBP (Tamborero et al., 2022) and PANDRUGS offer additional support, with PANDRUGS accommodating both gene and drug queries. In addition to guiding therapy selection at the individual level, broader approaches have been developed to analyze treatment trends across different tumor types at a larger scale (Rubio-Perez et al., 2015). While most of these methods prioritize drug selection based solely on somatic variants, germline variants also play a crucial role in drug metabolism, influencing treatment effectiveness and potential toxicity (Menden et al., 2018). As a result, patients may exhibit varied responses to the same therapy, ranging from high efficacy to ineffectiveness or even adverse drug reactions (ADRs). ADRs are significant contributors to morbidity and mortality and pose a financial burden on healthcare systems (Khalil and Huang, 2020). Variability in drug response primarily stems from genetic differences in genes encoding drug substrates or those involved in xenobiotic metabolism and transport (Roden et al., 2019).

To optimize drug selection based on germline variants, pharmacogenomic databases such as DrugBank (Wishart et al., 2018), PharmGKB (Whirl-Carrillo et al., 2021), and the Table of Pharmacogenomic Biomarkers in Drug Labeling (https://www.fda.gov/media/124784/) can be leveraged to prioritize effective drugs while avoiding those that may be ineffective or cause ADRs. Tools like PHARMCAT (Sangkuhl et al., 2020) enable the development of personalized treatments based on germline variants found in VCF files. Additionally, some platforms, such as MTBP, integrate both germline and somatic variant data for a more comprehensive approach to treatment selection.

Numerous knowledge bases and bioinformatics tools are available for variant annotation, biomarker identification, drug prioritization, and response prediction, serving as essential resources to help clinicians determine the best treatment options for their patients. One emerging biomarker in this field is tumor mutational burden (TMB), which has shown promise in identifying patients most likely to benefit from immunotherapy across various cancer types (Hellmann and Paz-Ares, 2018). TMB is determined by calculating the total number of somatic mutations per megabase (Mbp) of sequenced DNA. However, the lack of standardization in TMB assessment remains a challenge, limiting its universal applicability as a biomarker. While high TMB is generally linked to better immunotherapy responses, its predictive value is not consistent across all cancer types (McGrail et al., 2021). Tumor Mutational Burden (TMB) has emerged as a promising genomic biomarker for identifying patients likely to benefit from immunotherapy across various cancer types (Hellmann and Paz-Ares, 2018). However, a lack of standardization in TMB assessment complicates its use as a universally reliable biomarker. While high TMB is generally linked to better immunotherapy responses, its predictive value varies across different cancer types (McGrail et al., 2021). Advances in bioinformatics now enable in-depth TMB analysis, allowing for *in silico* hypothesis generation that goes beyond simple TMB-based patient stratification. By leveraging these insights, targeted therapies can be prioritized based on mutations with established treatment options. Bioinformatics advancements now allow for an in-depth analysis of TMB, generating *in silico* hypotheses that extend beyond simple TMB-based patient stratification. These insights can be used to prioritize targeted therapies based on mutations with known treatment options. For instance, PANDRUGS [24] is a platform that ranks drug treatments based on actionable mutations found in TMB, enabling a more precise approach to therapy selection.

Additionally, numerous bioinformatics tools and AI-based methodologies have been designed to facilitate the interpretation of cancer-related variants and suggest potential treatment options based on prior evidence (Cotto et al., 2018). These patient-centered resources rely on tumor-specific somatic variants and can be categorized based on the type of input data they require. For instance, if a list of variants is available, tools such as MTB-REPORT (Perera-Bel et al., 2018), the Cancer Genome Interpreter (CGI) (Tamborero et al., 2018), the Variant Interpretation for Cancer Consortium Meta-Knowledgebase (VICC METAKB) (Wagner et al., 2020), PREMEDKB (Yu et al., 2019), and the SMART Cancer Navigator (Warner et al., 2018) can provide useful insights. Some tools also accept disease- or drug-related queries, while those with access to a variant calling file (VCF) may utilize platforms such as MTBP (Tamborero et al., 2022) or PANDRUGS (Pineiro-Yanez et al., 2018), with the latter supporting both gene and drug queries.

Beyond individual patient analysis, large-scale approaches have been developed to guide treatment selection across various tumor types and identify broader trends in therapy response (Rubio-Perez et al., 2015). While many of these methods prioritize drug selection based on somatic variants alone, germline variants are equally important, as they play a crucial role in drug metabolism, effectiveness, and potential toxicity (Menden et al., 2018). This genetic variability leads to differing patient responses, ranging from positive therapeutic outcomes to ineffectiveness or even adverse drug reactions (ADRs). ADRs significantly contribute to morbidity, mortality, and increased healthcare costs (Khalil and Huang, 2020).

Differences in drug response are largely attributed to genetic variations in genes encoding drug substrates or those involved in xenobiotic metabolism and transport (Roden et al., 2019). By leveraging pharmacogenomic databases such as DrugBank (Wishart et al., 2018), PharmGKB (Whirl-Carrillo et al., 2021), and the Table of Pharmacogenomic Biomarkers in Drug Labeling (https://www.fda.gov/media/124784/), effective drugs can be prioritized over those that are ineffective or may cause ADRs. Tools like PHARMCAT (Sangkuhl et al., 2020) provide tailored treatment recommendations based on germline variants found in VCF files. Furthermore, platforms like MTBP integrate both germline and somatic variant data for a more comprehensive approach to personalized medicine (Borchert et al., 2021; Yao et al., 2020).

A crucial aspect of studying mutational events is the ability to differentiate significant mutations from those commonly found in the healthy population (Zarrei et al., 2015). Mutation detection approaches can be categorized into two main types: reference-free and reference-based methods (Raman et al., 2019). Reference-free methods normalize samples using inherent genomic features such as GC content and mappability, while reference-based tools rely on either a single normal sample matched to the sample of interest or a Panel of Normals (PON) (PON, 2021). The inclusion of normal samples helps eliminate variations introduced by experimental factors such as sample handling, preparation, and sequencing technology.

 structural variants, including those that cause copy-number variations (CNVs), can be highly complex in their impact on the genome (Schutte et al, 2019; Baca et al., 2013). Their characterization depends on various techniques, including paired-read and split-read analysis, as well as *de novo* genome assembly of the sample. However, the short read length of next-generation sequencing (NGS) imposes limitations on these analyses. The advent of advanced bioinformatics tools and long-read sequencing technologies has addressed these challenges, providing deeper insights into SVs (Cameron et al., 2021). Nanopore-based sequencers, for example, offer advantages such as portability and real-time data analysis. Additionally, bioinformatics tools facilitate the clinical characterization of SVs for diagnostic applications (Valle-Inclan et al., 2021).

SVs influence both germline and somatic genomic instability, contributing to disease development and potentially guiding therapy selection and drug response prediction. Some bioinformatics platforms designed for drug prioritization based on small variants also accept CNVs (Perera-Bel et al., 2018) and gene fusions (Wagner et al., 2020) as inputs. More sophisticated diagnostic approaches leverage shallow whole-genome sequencing (sWGS) for CNV analysis, aiming to establish CNV-based signatures that enable more precise diagnostics and treatment selection (Macintyre et al., 2018; van Belzen et al., 2021).

Mutational signatures in genomic DNA provide insights into the mutational processes driving cancer progression (Greenman et al., 2007; Degasperi et al., 2022). These signatures can be characterized by different mutation types, including single base substitutions (SBS), doublet base substitutions (DBS), insertions and deletions (indels), CNVs, and genomic rearrangements (Alexandrov et al., 2020. The identification of mutational signatures may aid in detecting therapeutically actionable biomarkers, supporting their use in personalized medicine. While over 30 mutational signatures have been identified, many remain of unknown origin. Some, however, have clear clinical relevance, such as those linked to tobacco exposure, ultraviolet (UV) radiation, and defects in DNA repair mechanisms, including mismatch repair and double-strand break repair. Studies have shown that tumors with DNA damage repair deficiencies exhibit therapeutic sensitivity to DNA-damaging agents and immunotherapy (Waddell et al., 2015; Ma J. et al., 2018; Ma X. et al., 2018; Connor et al., 2017). For example, a mutational signature associated with pathogenic BRCA1 and BRCA2 mutations in breast and ovarian cancers suggests homologous recombination (HR) deficiency, indicating sensitivity to PARP inhibitors (Lord and Ashworth, 2016) Conversely, prior exposure to DNA-damaging chemotherapy agents has been linked to drug resistance (Levatic et al., 2022). Mutational signatures also serve as molecular footprints of cancer therapies, helping estimate their contribution to tumor mutational burden (TMB) and revealing their long-term genomic effects (Pich et al., 2019). Tumor Mutational Burden (TMB) has emerged as a significant biomarker for predicting responses to immunotherapy across various cancer types. However, the assessment of TMB has faced challenges due to variability in measurement techniques, leading to efforts aimed at standardizing its evaluation. TMB measurement can vary significantly across different cancer types and sequencing platforms (Jardim et al., 2021). Various factors including differences in panel size, gene content, and bioinformatics pipelines contribute to this variability. For example, certain cancers like uterine, bladder, and colon cancers exhibit greater variability in panel TMB values compared to lung and head and neck cancers. This variability underscores the necessity for standardized methodologies to ensure consistent and reliable TMB assessment.​ To address these challenges, initiatives such as Friends of Cancer Research (Friends) TMB Harmonization Project is undertaken in which significant strides are made in unifying TMB measurement across various laboratories. By identifying approaches to enhance consistency in evaluating the genetic mutations of tumors, the initiative aims to improve the reliability of TMB as a biomarker (Merino et al., 2020) Various guidelines are proposed to harmonize TMB quantification across different diagnostic platforms. These recommendations focus on standardizing TMB reporting, aligning analytical validation studies, and ensuring consistent methodologies in clinical samples. Consistent measurement methodologies enable more accurate predictions of patient responses to immune checkpoint inhibitors, thereby informing treatment decisions across various cancer types (Huang et al., 2021). The standardized TMB evaluation facilitates the comparison of clinical trial results and supports the broader application of TMB as a predictive biomarker in oncology. (Sha et al., 2020) Computational methods for mutational signature analysis vary in their mathematical frameworks and fall into two main categories: *de novo* discovery of novel signatures and refitting methods for detecting known signatures (Baez-Ortega and Gori, 2019; Omichessan et al., 2019). Tools such as SIGPROFILER (Bergstrom et al., 2019; Islam et al., 2020; Bergstrom et al., 2020; Kim et al., 2016), previously used in the COSMIC database, and SIGNATUREANALYZER (Kim et al., 2016; Kasar et al., 2015; Haradhvala et al., 2018; Degasperi et al., 2020) were instrumental in analyzing large cancer genome datasets from PCAWG, TCGA, and ICGC projects. SIGNAL, a web-based tool, not only identifies mutational signatures but also links them to gene drivers, potentially revealing novel therapeutic dependencies (Degasperi et al., 2020). Additionally, HRDETECT predicts HR deficiency, helping to stratify patients based on their likely response to PARP inhibitors (Davies et al., 2017). In large-scale genomic studies such as the 100,000 Genomes Project, somatic variant data—including sequencing coverage, small variants, and structural variations—is visually represented using a Circos plot, offering an intuitive overview of genomic alterations (Srivastava, 2024a). Advancements in high-throughput technologies with ML based approaches have enabled the generation of large-scale human gut microbiota profiles, driving growing interest in uncovering the links between the gut microbiome and complex human diseases. Results indicated accuracy in identifying individuals at high risk by extracting and integrating insights from complex microbiome datasets with challenges in managing the heterogeneity and sparsity of microbial features and in capturing the underlying relationships among various human diseases (Huang et al., 2024). Data-tool such as scPriorGraph is used to construct biosemantic cell-cell graphs with prior gene set selection for cell type identification from scRNA-seq data (Cao et al., 2024).

## 3 Integrative multiomics strategies for drug selection

Advancements in high-throughput technologies have enabled the integration of multiple omics layers, providing a deeper understanding of biological systems (Hasin et al., 2017; do Valle et al., 2018). Tools such as PANOPLY (Kalari et al., 2018) and MOalmanac (Reardon et al., 2021) combine genomic and transcriptomic data to identify and prioritize potential drug targets. The Cancer Druggable Gene Atlas (TCDA) (Jiang et al., 2022) database compiles information on genomic alterations, including short variants, copy-number variations (CNVs), and gene fusions, along with gene expression, dependencies, and druggability.

DRUGCOMBOEXPLORER (Huang et al., 2019) incorporates DNA sequencing, gene copy number, methylation, and gene expression data from cancer patients to (a) identify key driver signaling pathways and (b) suggest effective anticancer drug combinations. Additionally, transcriptomic networks can be further enhanced with other omics layers, providing broader functional insights. For example, COSMOS (Dugourd et al., 2021) integrates phosphoproteomics, transcriptomics, and metabolomics to infer kinase and transcription factor activity. Deep learning algorithms are gaining popularity for multi-omics integration due to their ability to capture complex nonlinear and hierarchical relationships (Kang et al., 2022). One such tool, DEEPDRK (Wang et al., 2021), utilizes genomics, transcriptomics, epigenomics, and chemical compound properties to predict drug susceptibility in cancer cell lines and patients (Keskin et al., 2019).

Neoantigen prediction pipelines, such as PVACTOOLS (Hundal et al., 2020) incorporate computational tools to detect neoantigens from tumor DNA-seq and RNA-seq data. These tools also estimate an individual’s HLA class and rank neoantigens based on their molecular compatibility with the patient’s major histocompatibility complex (MHC) and other relevant parameters (Hackl et al., 2016). Furthermore, tools like CIBERSORTX (Newman et al., 2019) and MCP-COUNTER (Becht et al., 2016) analyze expression data to infer the presence of immune infiltrates in tumor tissue. Understanding the immune composition of a tumor, alongside tumor mutational burden (TMB) values, can aid in treatment selection. However, only a limited number of these tools currently prioritize drug treatments or neoantigen selection based on TMB content in clinical trials (Keskin et al., 2019). Intratumoural heterogeneity (ITH) within individual tumors is driven by a combination of somatic single nucleotide variants (SNVs), structural variations (SVs), transcriptomic and epigenetic modifications affecting gene expression, the tumor microenvironment (TME), and the antitumor immune response (Black and McGranahan, 2021; Nguyen TM. et al., 2021). ITH can be spatial, occurring in distinct tumor regions, or temporal, evolving over time through clonal progression. Understanding the extent of ITH and characterizing clonal subpopulations based on their unique mutational or transcriptomic profiles can be valuable for prioritizing drug treatments and predicting tumor response to therapy. This section provides an overview of key methodologies for dissecting ITH to guide drug selection.

A variety of user-friendly, web-based tools, such as Paintomics 4 (Liu et al. 2022), 3Omics (Kuo et al., 2013), and Galaxy (Galaxy, 2024), enable easy analysis with only a basic understanding of the underlying methodologies. More advanced tools, including integrOmics (Cao et al., 2009), SteinerNet (Tuncbag et al., 2012), Omics Integrator (Tuncbag et al., 2016), and MixOmics (Rohart et al. 2017), require programming expertise and offer customizable parameters for greater control over data analysis. Metabolomics datasets can be analyzed using the XCMSOnline (Tautenhahn et al., 2012) web tool, which integrates metabolomics data with genomic and proteomic information. A novel equivariant 3D-conditional diffusion model, called DiffFBDD, has been developed to generate new pharmaceutical compounds based on the 3D geometric structure of specific target protein pockets. DiffFBDD addresses the common underutilization of geometric information by leveraging an equivariant graph neural network to integrate detailed atomic-level data from protein pockets down to their backbone atoms (Zheng et al., 2025). AI-driven drug prioritization relies on a synergy between predictive modeling, network analysis, and knowledgebase integration, enabling personalized and data-driven therapeutic decision-making in oncology. Supervised learning models are widely used to correlate genomic alterations with drug response data. These models are trained on large pharmacogenomics datasets like GDSC (Genomics of Drug Sensitivity in Cancer) (Cokelaer et al., 2018) and CCLE (Cancer Cell Line Encyclopedia) to learn patterns between molecular features (e.g., gene expression, mutations) and drug sensitivity (Barretina et al., 2012). Deep learning based tools are particularly effective in integrating multi-layered omics data and capturing nonlinear interactions between genes, pathways, and drugs. Such as DeepDR (Jiang and Li, 2024), DeepSynergy (Preuer et al., 2018), and GraphDRP (Nguyen PHD. et al., 2021) utilize these architectures to predict drug response or synergistic drug combinations with higher accuracy. The drug prioritization scores can be computed using network-based methods, where biological networks (e.g., protein–protein interaction networks) are analyzed to identify key driver genes or pathways affected in a patient. These are then matched with known drug–target relationships using databases like DrugBank (https://go.drugbank.com/), DGIdb (Drug–Gene Interaction database) (https://dgidb.org/), and LINCS (Library of Integrated Network-Based Cellular Signatures) (Koleti et al., 2018). Other frameworks, such as OncoKB (Chakravarty et al., 2017), iCAGES (Dong et al., 2016), and PANOPLY (Mani et al., 2021), combine multiomics data with curated clinical and molecular knowledgebases to rank drugs based on patient-specific molecular alterations, mutation impact, and druggability. These tools not only improve treatment efficacy but also assist clinicians in identifying repurposable drugs and novel therapeutic strategies tailored to each patient’s molecular landscape. AI systems typically generate a drug prioritization score based on predicted sensitivity (e.g., IC50 or AUC values), Drug–target interactions and pathway relevance, Molecular similarity between tumor and drug response signatures and Integration of clinical trial or approved drug data (Paul et al., 2021). These scores are then ranked to help clinicians identify the most promising therapies tailored to an individual’s molecular cancer profile.

## 4 Genome profiling for tumor clonality

Tumors contain both clonal mutations, which are present in all cells, and subclonal mutations, which are restricted to specific subpopulations. The prevalence of subclonal mutations provides insight into tumor phylogeny, allowing researchers to identify active subclones and their evolutionary relationships. Cancer subclones undergo Darwinian evolution, where each subclone exhibits a distinct fitness level that can be inherited by daughter cells. Studies have shown that increased levels of CNVs may confer a selective advantage to certain subclones, enabling them to outcompete neighboring populations (Salehi et al., 2021).

Administration of anticancer drugs creates selective pressure that impacts subclonal fitness. Drug-sensitive cells are eliminated, but some subclones—often a minority—may acquire resistance through pre-existing mutations or *de novo* drug-induced mutations in drug-tolerant cells. These resistant subclones can subsequently expand, leading to tumor relapse. For example, research by Xie et al. identified a subgroup of quiescent glioblastoma cancer stem cells (CSCs) that survived antiproliferative chemotherapy, later re-entered the cell cycle, and contributed to tumor regrowth, ultimately causing treatment failure and relapse (Xie et al., 2022). Other studies have suggested combining multiregion sampling to analyze spatial ITH with the monitoring of circulating tumor DNA (ctDNA) through liquid biopsies to track clonal evolution in real time and adjust therapies accordingly (Amirouchene-Angelozzi et al., 2017; Siravegna et al., 2017). A Bayesian evolutionary framework has also been applied to investigate the spatiotemporal dynamics of cancer subclones within individual patients (Alves et al., 2019).

Subclone identification can be performed using various approaches, including genome profiling and single-cell sequencing. Genome profiling remains the primary strategy for studying clonal evolution. Several bioinformatics tools have been developed to infer cancer subclones based on SNV allele frequencies, CNV profiles, and tumor purity measures, including PYCLONE-VI (Gillis and Roth, 2020), PHYLOWGS (Deshwar et al., 2015), FASTCLONE (Xiao et al., 2020), SCICLONE (Miller et al., 2014), and MOBSTER (Caravagna et al., 2020). However, this approach has limitations. It primarily detects mutations present in most or all tumor cells, while stromal contamination can influence mutation frequency estimates. Additionally, many prior inference steps in these tools may introduce errors, which can propagate through subsequent analyses (Turajlic et al., 2019).

The concept of clonetherapy has emerged, aiming to optimize treatment regimens that account for ITH by targeting all subclones, including minor populations with relapse potential (Jiménez-Santos et al., 2022). Several computational tools support this approach, such as OmicsTIDE (Harbig, 2023), which enables interactive exploration of multi-omics data trends; FORALL, (Aswad and Jafari, 2023), an interactive Shiny/R web portal for navigating high-throughput multi-omics data in pediatric acute lymphoblastic leukemia; MMDRP (Taj and Stein, 2024) which applies multi-modal deep learning for drug response prediction and biomarker discovery; and iCluF (Shakyawar et al., 2024), an unsupervised iterative cluster-fusion method for patient stratification using multi-omics data.

## 5 Incorporating drug prioritisation tools into the clinical practice

**FIGURE 2:**
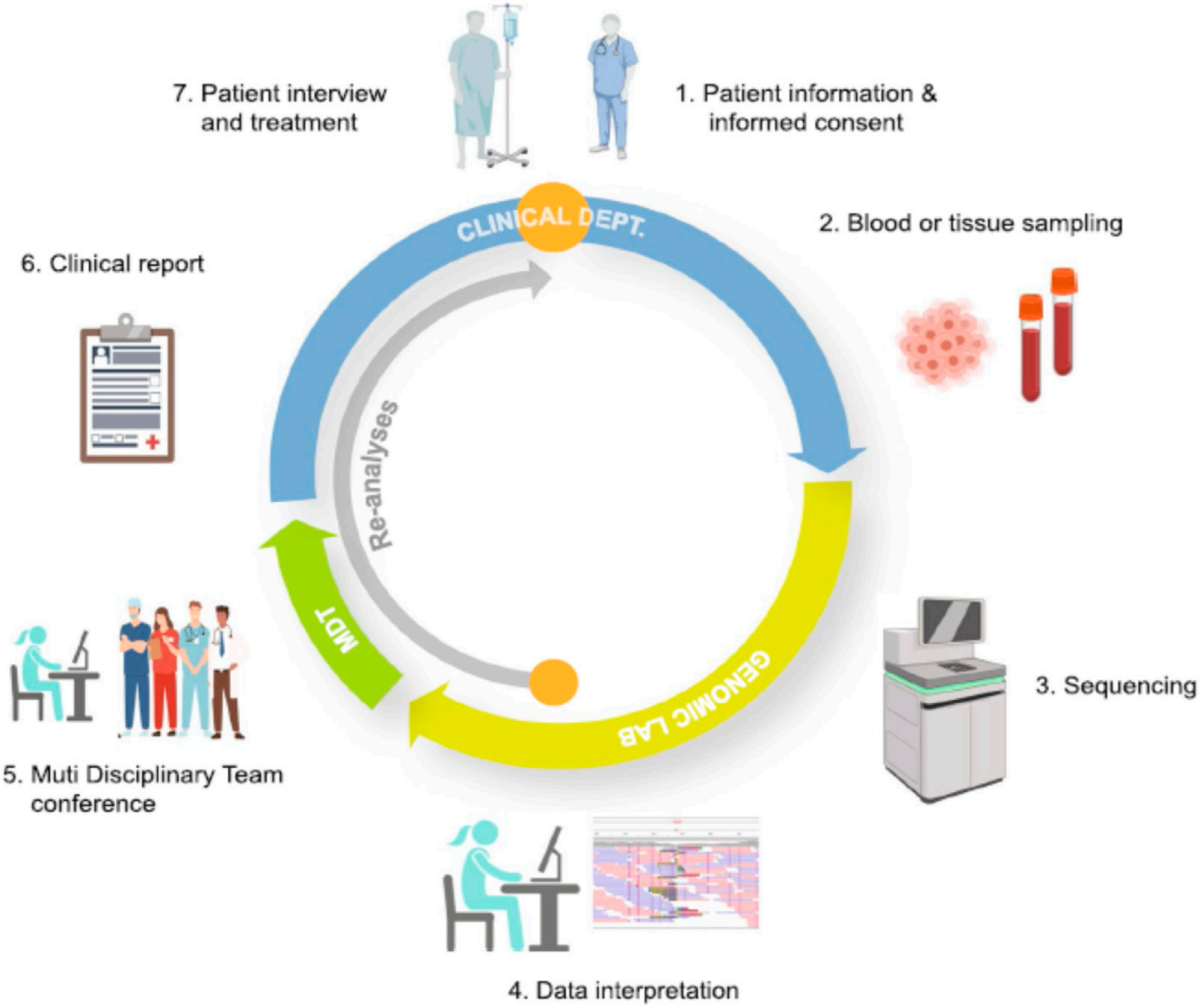
Whole Genome Sequencing (WGS) from Patient to Clinical Report-WGS could provide valuable clinical insights—either by confirming a diagnosis or suggesting alternative treatment options. After patient consent, a sample of whole blood or tumor tissue is sent to a specialized laboratory equipped for WGS. The skilled professionals meticulously analyze the sequence data. A multidisciplinary team (MDT) establish a definitive diagnosis and evaluate the clinical significance of the detected variants. Once finalized, the clinical report is reviewed by attending physician and the results are discussed with the patient, which includes the implications of the findings, their impact on the patient’s condition, and recommended next steps. If the initial analysis does not identify a disease-causing variant, the stored WGS data is periodically re-analyzed (inner grey arrow). The continuous process allows for the incorporation of new scientific discoveries, potentially leading to a diagnosis without requiring further hospitalization or additional sampling. Additionally, other clinically relevant insights, such as pharmacogenetic data can be extracted from the WGS data to enhance patient care. This figure is adapted from Bagger, FO. et al. BMC Med Genomics 17, 39 (2024).

Bioinformatics-driven therapy selection remains in its early stages, with drug prioritization methods still facing significant technical and biological challenges that hinder their routine clinical application. However, considerable progress has been made to integrate these methodologies into medical practice for patient benefit. Cancer care spans multiple stages, from disease prevention and early detection to diagnosis, treatment, and follow-up. To determine the most effective treatment options, physicians require integrated patient information presented in a clear and interpretable format through clinical decision support systems. These systems must efficiently access electronic medical records containing diverse data types, including genomic information collected at different stages of a patient’s journey. See Figure 2.

Next-generation sequencing (NGS) data analysis, including drug prioritization algorithms, will be incorporated into clinical decision support systems, necessitating broad interoperability across data, metadata, research software, and computational infrastructure. This requires standardized nomenclature, well-annotated genomic datasets linked to clinicopathological information, and efficient data-sharing mechanisms. To achieve this, multimodal cancer data must be meaningfully integrated, highlighting the importance of data harmonization and standardization. Several initiatives are actively addressing this challenge. The Findable, Accessible, Interoperable, and Reusable (FAIR) principles facilitate efficient clinical data exchange (Kush et al., 2020). Data harmonization efforts include the NIH Data Commons (https://commonfund.nih.gov/commons) and the Cancer Research Data Commons (CRDC) (https://datacommons.cancer.gov/). Additionally, ICGC-ARGO (https://platform.icgc-argo.org/) is working to collect comprehensive cancer genomic datasets enriched with clinical information, health records, and treatment response data (ICGC, 2022). The Beyond 1 Million Genomes (B1MG) initiative is also advancing efforts in this direction. The integration of multiomics approaches and *in silico* drug prioritization tools into routine clinical practice requires continued development within healthcare systems. These tools would significantly benefit from extensive, standardized clinical, pathological, and genomic annotations within a federated data-sharing model that preserves patient privacy while storing retrospective treatment response data. Such a framework would facilitate benchmarking, training, and validation of novel drug response prediction models and support the identification of new predictive biomarkers based on historical data (Rajpurkar et al., 2022). The Global Alliance for Genomics and Health (GA4GH) provides international policies and standards to ensure responsible access to genomic and health-related data (Rehm et al., 2021). Projects such as the GA4GH Genome Beacons have pioneered bioinformatics frameworks that allow hospitals to query clinicogenomic datasets while maintaining data privacy and ownership (Fiume et al., 2019).

Artificial Intelligence (AI)-driven Clinical Decision Support Systems (CDSS) have been implemented in various real-world medical settings, demonstrating their potential to enhance diagnostic accuracy, streamline workflows, and improve patient outcomes. The notable case studies include Instant Skin Cancer Diagnosis in NHS Hospitals, at Chelsea and Westminster Hospital in London, an AI tool named ‘Derm’ is being utilized for rapid, autonomous skin cancer assessments. Healthcare professionals use an iPhone equipped with a magnifying lens to capture images of suspicious moles, which the AI app analyzes within seconds. The ‘Derm’ system boasts a 99.9% accuracy rate in ruling out melanoma and has significantly reduced waiting lists by enabling doctors to focus on more severe cases. Currently adopted by 20 NHS hospitals, this technology has detected approximately 13,000 cancer cases to date. (https://www.chelwest.nhs.uk/about-us/news/chelsea-and-westminster-hospital-leads-the-way-with-autonomous-ai-technology-to-speed-up-life-saving-skin-cancer-checks, 2025) In another study, AI Assisted in Radiology Diagnoses in South Australia, South Australian Medical Imaging (SAMI) has integrated AI to assist in interpreting chest X-rays across multiple hospitals. This AI functions as a “spell checker” for radiologists, highlighting areas of interest and suggesting potential diagnoses. SAMI performs approximately 700,000 radiological examinations annually (https://www.sahealth.sa.gov.au/). Enhanced Cancer Detection Rates in General Practices has been carried out in England, in which the ‘C the Signs’ AI tool has been deployed in around 1,400 general practices to analyze patient medical records for hidden patterns indicative of cancer risk. Its implementation led to an increase in cancer detection rates from 58.7% to 66.0% with identification of over 50 different types of cancer, ensuring faster and earlier diagnoses (Bakshi et al., 2024). A regional hospital implemented an AI-based CDSS aimed at reducing 30-day all-cause hospital readmission rates. The AI tool combined clinical and non-clinical data to predict patients’ risk of readmission and provided recommendations to mitigate this risk (Romero-Brufau et al., 2020). Another study involving the Watson for Oncology (WfO) AI-based CDSS assessed its influence on treatment decisions for complex breast cancer cases (Xu et al., 2020). These case studies illustrate the tangible benefits and effectiveness of AI-driven CDSS in diverse medical environments, highlighting their role in improving diagnostic accuracy, patient management, and overall healthcare delivery.

## 6 Challenges

The process of drug prioritization in cancer research is hindered by several biological and technical challenges. A major obstacle is the shortage of experts with specialized knowledge in multiomics analysis, bioinformatics, and clinical interpretation. Furthermore, the accessibility and availability of clinical samples remain problematic, exacerbated by the lack of standardized protocols for sample processing, which can result in inconsistencies in data quality and reliability. Scalability is another pressing issue, as translating multiomics findings into clinical applications requires a robust infrastructure capable of managing large-scale data generation and analysis. The absence of standardized, high-quality reference datasets for training and validating genomic analysis methods further complicates efforts to ensure accuracy and reproducibility. Additionally, many healthcare institutions face computational limitations, making it difficult to efficiently process and integrate large-scale omics data. Strict data privacy regulations add another layer of complexity, as maintaining patient data security and confidentiality is crucial. AI-driven genomics offers powerful tools for precision oncology, but it also faces several technical and computational challenges. A key concern is model interpretability. Various deep learning models function as “black boxes,” making it difficult to understand how specific features influence predictions, which is critical in clinical settings. Additionally, data heterogeneity across omics platforms, patient populations, and sequencing technologies complicates data integration and can lead to inconsistent results. Biases in AI models, often stemming from imbalanced training data or underrepresented subpopulations, can result in skewed predictions that may not generalize well across diverse patient groups. Addressing these challenges is essential for building reliable, equitable, and clinically useful AI tools in genomics (Dias and Torkamani, 2019).

Moreover, the ethical and legal implications of using omics data in clinical settings must be carefully addressed to establish a comprehensive regulatory framework. While implementing AI-driven genomics tools in precision oncology, several ethical, legal, and social issues must be addressed to ensure safe, fair, and responsible use (Farasati, 2023). These issues are the Data Ownership, Informed Consent, Algorithmic Bias, Transparency and Accountability, and Data Privacy and Security. The Patients often do not have direct control over their genomic data once it is collected, especially when stored in centralized or commercial databases, due to Institutional vs. personal ownership. Further lack of standardization around who can access, use, or profit from genomic data can hinder trust and data-sharing (Gerke et al., 2020). Patients may not fully understand how their data will be analyzed, integrated, or reused over time. The one-time consent forms are not sufficient for evolving AI applications. The patients may not have consented to secondary use for unrelated research or algorithm training. AI models trained on predominantly western, caucasian, or male genomic datasets which may yield inaccurate predictions for diverse populations. Sometimes there are biased outcome predictions (Cross et al., 2024). Algorithms may perform well in controlled settings but fail in real-world, and heterogeneous clinical populations. Many models, particularly deep learning networks lack explainability, making it difficult for clinicians and patients to trust decisions. The guidelines for validation, certification, or clinical approval of AI-driven genomics tools are still emerging. The genomic databases are high-value targets for breaches, with implications for patient confidentiality and potential misuse (Bonomi et al., 2020). Even de-identified genomic data can be re-identified due to its unique nature. And most importantly, the accountability for errors remains unclear (Martinez-Martin and Magnus, 2019).

Biologically, a key challenge lies in the incomplete understanding of inter and intratumor heterogeneity and the somatic evolutionary processes driving cancer progression. The relationship between clonal expansion and cancer initiation remains unclear, as do the intricate topological interactions between tumors and the tumor microenvironment (TME), including cell–cell communication. Another critical issue is the progressive exhaustion of antitumor immunity, which limits therapeutic efficacy. Furthermore, the mechanisms underlying the emergence and expansion of drug-resistant subclones have not been fully elucidated. There is also a lack of comprehensive characterization of genetic and epigenetic alterations—such as structural variations, and transcriptional driver mutations—and their impact on drug response. The interplay between aging, cellular senescence, and drug efficacy remains poorly understood, further complicating treatment approaches. Additionally, information on the influence of germline variants on adverse drug reactions (ADRs) in many anticancer therapies is still insufficient.

On the technical side, the use of formalin-fixed, paraffin-embedded (FFPE) sample preparation can cause DNA fragmentation and degradation, making it difficult to distinguish true variants from artifacts in genomic analyses. Another challenge is the trade-off between sequencing scope and read depth—whole genome sequencing (WGS) provides a broad view but at lower coverage, whereas targeted sequencing offers deeper reads but a narrower scope. Similarly, in single-cell sequencing, increasing the number of cells analyzed reduces the read depth per cell. Multi-alignment reads pose difficulties due to repetitive genomic regions, complicating accurate variant calling. While short-read sequencing is widely used, it struggles to detect large structural variations, whereas long-read sequencing, despite its ability to identify these variations, has a higher error rate. Additionally, the lack of standardized guidelines for analyzing spatial data in single-cell technologies presents a significant challenge. Finally, predicting toxic interactions and synergistic effects in combination therapies remains a major hurdle in optimizing cancer treatment strategies. These computational, biological and technical limitations collectively hinder the accurate prioritization of drugs for cancer treatment, highlighting the need for continued advancements in genomics, immunology, and computational biology. Addressing these challenges is essential for the successful integration of multiomics approaches into personalized medicine and routine patient care. Numerous studies have shown that AI can surpass human capabilities in interpreting the vast amounts of data associated with complex diseases like cancer. However, AI should be seen as a tool to enhance human intelligence rather than replace it. Any analysis conducted by AI must be reviewed and validated by domain experts. Additionally, machine learning (ML) and deep learning (DL) models require oversight from specialists in bioinformatics and programming to ensure their reliability and accuracy.

One of the primary challenges in applying AI and DL to cancer diagnosis, prognosis, and treatment is the “black box” problem. This refers to the lack of transparency regarding how AI systems process information and arrives at conclusions. When AI operates autonomously with minimal human oversight, it may become unclear how it selects features or makes decisions, potentially leading to skepticism about its predictions. This uncertainty could force clinicians and researchers to accept AI-generated results on “blind faith” (Sorell et al., 2021). In response, researchers have been working to develop AI systems that provide explainable insights for physicians and clinicians. To ensure data availability and sharing while protecting patient privacy in multiomics cancer data, several technical and strategic approaches are employed such as Federated Learning (FL) (Saha et al., 2024), Differential Privacy (DP), Secure Multi-Party Computation (SMPC) (Zhou et al., 2024), Homomorphic Encryption (Ogburn et al., 2013), Trusted Research Environments (TREs) (Kavianpour et al., 2022), Data De-identification and Anonymization (Chevrier et al., 2019), Synthetic Data Generation, Standardized Data Use Agreements (DUAs), Dynamic and Informed Consent Models (Wendland et al., 2022) and Adherence to FAIR and CARE Principles (Carroll et al., 2021). These approaches collectively support secure, ethical, and effective sharing of multiomics cancer data, facilitating advances in research and personalized medicine. For example, Kwong et al. (2022) designed an AI model using ML to predict whether prostate cancer patients would benefit from nerve-sparing radical prostatectomy by assessing the likelihood of tumor extension beyond the prostate. The AI’s decision-making process was made interpretable using a publicly available web application, Shapley Additive exPlanations (SHAP) (Kwong et al., 2022). Deep learning also requires vast amounts of data to develop robust algorithms applicable to new datasets. Consequently, cancer research studies must collect multiple samples to serve as training data (Hussain et al., 2017). Furthermore, the use of AI and big data raises ethical concerns, particularly regarding patient data privacy. In some cases, patient data is used for purposes beyond direct medical care, and this may occur without the patient’s explicit consent (Rigby, 2019). A significant challenge in multiomics integration is missing data, as not all biomolecules are measured across all samples. This can be due to financial constraints, instrument sensitivity, or other experimental limitations, leading to incomplete datasets for certain omics technologies. While recent advancements in AI and statistical learning have greatly improved multiomics data analysis, many techniques still assume the presence of fully observed data. However, new approaches are being developed to address the issue of missing data, allowing for more effective utilization of incomplete datasets and paving the way for improved precision oncology in the future. Handling missing values in multi-omics data is a critical step for improving the accuracy and robustness of downstream analysis, as such datasets often suffer from incomplete measurements across different omics layers due to technical limitations or sample variability. Several specific methods and tools have been developed to address this challenge effectively such as Statistical Imputation Techniques, Matrix Factorization Methods, Bayesian and Probabilistic Models, Machine Learning-Based Imputation and Deep Learning Methods (Huang et al., 2023). There are various Multi-Omics-Specific Tools such as MOFA (Multi-Omics Factor Analysis) (Hama et al., 2023) and MAGIC (Markov Affinity-based Graph Imputation of Cells (van Dijk et al., 2018) and impute omics designed for imputing missing values in multiomics datasets using joint matrix completion and feature correlations across omics types. Methods like multi-view learning and tensor factorization integrate data from multiple omics layers simultaneously, enabling imputation that leverages cross-omics relationships. Thus selecting an appropriate imputation method depends on the extent and pattern of missingness, the data type (e.g., continuous vs. categorical), and the structure of the dataset. More advanced, multiomics-aware approaches especially those based on probabilistic modeling and deep learning are increasingly favored for their ability to preserve biological signals and improve the accuracy of downstream analyses such as clustering, classification, and biomarker discovery (Jadhav et al., 2019).

## 7 Future prospects

The advancement of bioinformatics tools and platforms will be essential for the future of multiomics research. These tools must enable seamless integration and analysis of diverse omics datasets, including genomics, transcriptomics, proteomics, and metabolomics. Enhancements in computational power, data storage, and cloud computing will further support large-scale multi-omics data processing. Additionally, blockchain technology presents a promising solution for data management, ensuring integrity, security, and patient privacy. By providing a transparent and tamper-proof system for storing and sharing multi-omics data, blockchain can help build trust and encourage collaboration within the healthcare community. The future of AI-driven bioinformatics in cancer treatment will likely involve increased interdisciplinary collaboration, drawing from bioinformatics, systems biology, computational biology, and clinical research. These collaborative efforts will be instrumental in addressing complex biological questions and developing comprehensive disease models. Strong partnerships between data scientists and clinicians will also help bridge the gap between intricate multi-omics data analysis and its practical applications in healthcare, ensuring that insights are both clinically relevant and actionable.

Despite its potential, multi-omics research still faces several challenges that must be overcome to fully realize its benefits. One key issue is the standardization of data collection and analysis across different omics layers and research institutions. Establishing standardized protocols and quality control measures will be critical to ensuring the reliability and reproducibility of multi-omics studies. Additionally, the complexity and high dimensionality of multi-omics data necessitate the development of advanced statistical methods and sophisticated algorithms for accurate interpretation and meaningful conclusions.

Multiomics approaches are expected to play a pivotal role in the advancement of personalized medicine. By integrating genetic, transcriptomic, proteomic, and metabolomic data, healthcare providers can tailor treatments to each patient’s unique characteristics, improving outcomes and minimizing adverse effects. Whole genome sequencing (WGS) and whole exome sequencing (WES) also hold great promise for enhancing our understanding of complex diseases such as cancer, cardiovascular conditions, and neurodegenerative disorders. By uncovering the molecular mechanisms driving these diseases, multiomics studies can help identify novel biomarkers for early diagnosis and potential drug targets. Over the next decade, significant advancements in multiomics integration with other emerging technologies could lead to the development of personalized virtual models of patients. These models would allow for *in silico* testing of treatments and interventions before their application in real-life clinical settings. Additionally, improvements in multiomics data visualization tools will enhance researchers’ and clinicians’ ability to interpret complex datasets, facilitating the translation of AI-driven genomic insights into clinical practice.

The future of AI in genomics is promising, with numerous emerging trends, technological breakthroughs, and interdisciplinary approaches driving innovation in precision medicine. Addressing current challenges and exploring new applications will be essential for unlocking the full potential of this research. Continued investment, along with collaborative efforts across various fields, will ensure that AI-powered genomics remains at the forefront of scientific and medical progress.

## 8 Conclusion

The integration of intelligent computing in genomics for cancer research represents a crucial step toward unlocking the full potential of precision medicine. With approximately 14 billion laboratory tests conducted annually, clinical laboratories contribute to nearly 70% of medical decisions, highlighting the necessity for accurate and comprehensive data. The incorporation of AI in developing health indices, predicting health trajectories, and combining advanced statistical modeling with digital twins (DTs) showcases the transformative potential of these technologies in revolutionizing healthcare delivery. However, overcoming key challenges—such as generating actionable and concise metrics from omics data and establishing meaningful intra-level comparators—is essential for progress. As these advancements reshape the medical landscape, ethical considerations remain paramount to ensuring that technology complements, rather than replaces, the human touch in healthcare. The future of medicine depends on a deep understanding of patient journeys and care pathways, ensuring that AI-driven innovations align seamlessly with the complexities of individual wellbeing while enhancing patient-centered care.
